# The Impact of Genotype and Age on Energy and Protein Utilization in Individually Housed Brown Laying Hens

**DOI:** 10.3390/ani11123508

**Published:** 2021-12-09

**Authors:** Frederik J. Kleyn, Peter Vincent Chrystal, Mariana Ciacciariello

**Affiliations:** 1 Spesfeed Consulting (Pty) Ltd., P.O. Box 955, Broederstroom 0240, South Africa; 2 Department of Animal and Poultry Science, University of KwaZulu-Natal, Private Bag X01, Scottsville 3209, South Africa; CiaccM@ukzn.ac.za; 3 Complete Feed Solutions, Cockle Bay 2145, New Zealand; peter@completefeeds.co.nz

**Keywords:** age, efficiency, energy, genotype, hen, lysine

## Abstract

**Simple Summary:**

An investigation into whether the nutrient requirement of egg-laying hens has changed with genotype improvements was conducted. This study compared the response of individually housed laying hens of two different genotypes and ages. The strains used were a heritage breed in mid-lay and a modern breed at peak production. Energy was utilized with the same efficiency by both strains. All hens were able to adjust their feed intake to ensure that their energy requirements were met. The modern strain utilized protein slightly more efficiently than the heritage strain. It is unlikely that the nutrient requirements of modern layer strains have increased. More likely, requirements have decreased because modern hens are lighter and have a lower daily egg output (lay smaller eggs) despite their higher lifetime egg output. Regardless, feeding programs need to be adjusted for economic reasons and depend on achievable feed intakes under particular circumstances.

**Abstract:**

Responses to dietary energy and protein levels were compared between two egg-laying genotypes. Individually housed hens of a historic strain (HS) and a modern strain (MS) were compared. In Experiment 1 (Exp.1), four levels of true metabolizable energy, corrected for zero nitrogen retention (TME_n_) and four levels of total lysine, were offered from 30 to 40 weeks of age. In Experiment 2 (Exp.2), three levels of apparent metabolizable energy, corrected for zero nitrogen retention (AME_n_) and four levels of standardized ileal digestible lysine (SID Lys), were fed from 20 to 30 weeks of age. A randomized factorial block design (4 × 4 Exp.1 and 3 × 4 Exp.2) was applied. Energy utilization for egg output (EO) did not differ (NS), and both strains maintained a constant kJ intake (NS). The efficiency of SID Lys utilization for EO differed, with the MS being the more efficient (*p* < 0.034). A single model could be used to predict feed intake, using BW, EO, AME_n_ and SID Lys (r = 0.716). In conclusion, it is unlikely that the requirements of modern layer strains have increased. However, feeding programs should be adjusted for economic reasons and are dependent upon achievable feed intake under particular circumstances.

## 1. Introduction

In recent decades, the genetic potential of laying hens has improved substantially. The number of eggs produced has increased from 216 eggs per year in 1960 [1] to 325 eggs per year in 2019 [2], representing an increase from around 60% hen day (HD) production to nearly 90%. The improvement was achieved through the use of phenotypic selection, linear models and genomic selection tools. The pre-eminent selection criterion is improved persistency by selecting individual hens that lay longer clutches of eggs and a decline in the number of birds that never come into production [3,4]. Mature body weight and individual mean egg size have declined. Selection for more uniform egg size has increased early egg weights and reduced egg size late in lay [2,5,6]. The feed conversion ratio (FCR) per kg of eggs has improved from 3.44 in 1960 [1] to less than 2.00 currently [2].

Comparative work assessing the efficiency of protein and energy utilization in different poultry genotypes has been carried out. The efficiency of protein utilization (above maintenance) was similar in both fast-growing (broilers) and slow-growing genotypes (cockerels from an egg-laying strain) [7]. The percentage of nitrogen retained did not differ between broilers chickens and Leghorn hens (laying stock), with broilers exhibiting 3 to 4% lower metabolizability of energy than Leghorns. This implies that broilers have increased energy losses in faeces and urine [8,9]. Broilers and layer pullets did not show significant differences in AME_n_ utilization at either 9 or 21 days of age [9]. This body of work has demonstrated that protein utilization by diverse genotypes and ages of chicken remains essentially unchanged. Differences in the metabolizability of energy are moderate.

Some comparative work on evolving broiler genotypes’ growth and nutrient requirements has been published [10,11], but similar studies do not appear to exist for laying hens. It is intimated that the nutrient requirements of modern genotypes have increased [12,13,14,15]. Kidd and Loar [16] point out that the rate of change in the egg industry is such that it exceeds the science required from research to support emerging management practices. As laying hens evolve, hens’ amino acid (AA) nutritional needs will need to be reassessed.

An opportunity arose to investigate the response of individual laying hens, of two different genotypes and ages, to increases in dietary balanced protein and energy. Two experiments were conducted three decades apart, but the hens were housed in the same facility, and a similar experimental design was applied. The hens used were the Hisex Brown, a historic strain (HS) evaluated in 1986 as reported by Kleyn [17], and the Hy-Line Brown, a modern strain (MS) evaluated in 2018. While reviewing how layer genotypes have evolved is of interest, the fundamental objective of this body of work was to investigate the requirements and efficiencies of utilization of protein and energy by flocks differing in age and genotype. The objective of this study was to confirm whether modern layer genotypes’ nutrient and energy requirements have changed and guide feeding programs for modern genotypes.

## 2. Materials and Methods

Experiment 2 was approved by the Animal Ethics Committee of the University of KwaZulu-Natal (AREC/044/017). Birds were handled within the South African *Poult.* Association’s code of conduct (SAPA, 2018). Neither the ethics committee nor the SAPA code existed when Exp.1 was conducted. Each study investigated the response to differing energy and balanced protein levels on several production parameters. Two experiments, each of 10 weeks duration, were conducted in an open-sided convection house where individual laying hens could be accommodated and fed separately. Feed and water supply was *ad libitum*. Photoperiod was maintained at a constant 16 h/d by artificial lighting. It was not possible to control the environment. control

Of necessity, two different time frames, three decades apart, were used, but the birds were housed in the same facility, using a similar experimental design.

In Exp.1, 28-week-old HS hens were obtained from a commercial producer and placed in the experimental facility. In Exp.2, day-old MS chicks were obtained from a commercial hatchery and reared according to the primary breeder guidelines. At 19 weeks of age, the hens were placed and given two weeks to adapt to their new housing. Hens were individually housed in wire cages (500 mm depth × 450 mm height × 350 mm width). Each dietary treatment was randomly allocated to 12 replicate cages in Exp.1 and 16 replicate cages in Exp.2.

Experimental diets were fed for six weeks before data collection began to ensure the hens had depleted any possible reserves, thus reducing the potential effect of any previous feeding or management regime. Data were collected over the final four weeks. Hen day production, egg weight (EW) (g) and voluntary FI (g) were determined weekly, whereas BW (g) was measured at the start and end of the data collection period. The change in BW (g/d) was calculated for this period. Egg output (EO) was calculated as the product of EW × HD (g egg/hen/d), and FCR (g feed/g egg) was calculated as the ratio of average daily FI (g) to EO (g/d).

### 2.1. Experiment 1

In Exp.1, 192 HS hens were offered test diets from 30 to 40 weeks post-hatch. Four levels of true metabolizable energy, corrected for zero nitrogen retention (TME_n_) and four levels of total Lys were offered. A completely randomized 4 × 4 factorial block design was used, with 12 replicates per treatment. Diets were formulated using typical commercial practices in 1986, together with feed ingredients available at the time. Four basal mash diets were formulated and then mixed by a commercial feed supplier (Table 1). Although records were not retained, it is standard practice in South Africa to use a 6.0 mm hammer-mill screen when manufacturing layer mash. When the experiment was conducted, AA digestibility was not yet a consideration. Thus, the reference AA was total Lys, but all feeds were formulated to contain an ideal AA profile (Table 2). The basal diets were blended on-site to provide sixteen dietary treatments (Table 3).

Feed analysis was undertaken by the laboratory of the University of KwaZulu-Natal. Crude protein was determined in a LECO FP2000 Nitrogen Analyser (Leco Corporation, St Josephs, MI, USA) using the Dumas Combustion method. In order to determine Ca, samples were wet ashed and levels determined using a Varian Spectra AA-200 Atomic Absorption Spectrophotometer (Varian Inc., Palo Alto, CA, USA). Samples were digested with sulphuric acid, hydrogen peroxide and a selenium catalyst and then analysed for phosphorus using a Technicon Autoanalyser II (Technicon Inc., Mequon, WI, USA). All AA were analysed on a Beckman Amino Acid Analyser System 6300 (Beckman Instrumants, Inc., Fullerton, CA, USA), while TMEn was measured as described by McNab and Fisher [18].

For comparative purposes, the nutrient composition of the diets utilized in Exp.1 was recalculated using the same ingredient matrix values used to formulate the diets in Exp.2. This was achieved by the simple expedient of using a standard feed formulation matrix for both experiments and then recalculating the nutrient profiles of the diets used in Exp.1. This was imperative for a comparison of this nature because only total AA and TME_n_ values were used for the original feed formulations. The values for apparent metabolizable energy, corrected for zero nitrogen retention (AME_n_) and standardized ileal digestible lysine (SID Lys), were recalculated and then used for comparative purposes (Table 2).

### 2.2. Experiment 2

In Exp.2, 192 MS hens were studied from 20 to 30 weeks post-hatch. In this instance, three levels of dietary AME_n_ and four levels of SID Lys were offered to hens at peak production. A completely randomized 3 × 4 factorial block design was used, with 16 replicates per treatment. Diets were formulated using typical commercial feed ingredients, and within limits, similar ingredient content was maintained in all diets. Prior to diet formulation, yellow maize, wheat middlings, soybean meal and sunflower oilcake meal were characterized by near-infrared spectroscopy. Four basal mash diets were formulated to provide two levels of AME_n_ × two levels of dietary SID Lys (Table 4 and Table 5) and then mixed using coarsely ground maize (6.0 mm hammer-mill screen). A specialist laboratory feed supplier mixed the basal diets, then blended on-site to provide twelve dietary treatments (Table 6). All diets were formulated using SID Lys as the reference AA but contained the same ideal AA profile (Table 5). Feed analysis was undertaken by Evonik Africa (Pty) Ltd., using wet chemistry for CP protein and AA. AME_n_ was determined using AMINONir^®^ NRG, a methodology based on the determination of AME_n_ using the WPSA prediction equations [19]. No in vivo AA digestibility work was conducted as it was assumed that the values derived from NIR analysis were adequate.

### 2.3. Statistical Analysis

Data were analysed by full factorial ANOVA using JMP^®^ Pro 14.2.0. (SAS Institute Inc., Cary, NC, USA). Differences among treatment means were detected using Fisher’s protected least significant difference test at *p* < 0.05. Other relationships, where appropriate, were determined using multiple-linear regression, Pearson’s correlations and Student’s *t*-test pair-wise comparisons. Any mortalities or hens that did not lay an egg were treated as missing plots.

### 2.4. Comparison between Experiments

When comparing the two experiments, any hen that fell outside the specified FCR range of 1.5 to 2.4 was excluded [19,20]. This resulted in the exclusion of 25.5% of the hens in Exp.1 and 5.2% of the hens in Exp.2. The objective of this practice was to reduce the effect of body protein and energy on deposition or mobilization and minimize the impact on SID Lys and AME_n_ utilization. It also served as a mechanism to eliminate those birds that were inherently inferior producers.

Analysis of variance was performed to highlight the differences between the two flocks of hens. Linear regression prediction equations were used to evaluate and compare the two data sets, with each hen representing a single data point.

Energy was considered first. The AME_n_ intake, EO response to AME_n_ intake and the efficiency of energy utilization were determined (Equations (1) to (4)) (Table 7). The efficiency of AME_n_ utilization was determined as follows: (EO (g/d) × 9.157)/AME_n_ intake (kJ/d) × 100. This relationship is based on the assumption that each gram of egg required 9.157 kJ AME_n_ (Equation (1)). Body weight was used rather than metabolic body weight, as the latter did not improve the fit of any of the models. The SID Lys intake, EO, level in the diet, and the efficiency of utilization were determined (Equation (7) to (10)) (Table 8). The efficiency of SID Lys utilization (expressed as a percentage) for egg production was calculated as suggested by Spek [21] as follows: (EO (g/d) × 9.3)/SID Lys intake (mg/d) × 100. This relationship is based on the contention that 1 g of EO contains 9.3 mg Lys. These relationships were used on the basis of the comparison between the two flocks.

## 3. Results

### 3.1. Experiment 1

The analysed composition of the four basal diets used in Exp.1 is shown in Table 2. It was found that analysed values for both the Lys and TME_n_ levels were in close agreement with the formulated values. However, the determined methionine values were below expectation.

A detailed analysis of the average performance data obtained for the period 37 to 40 weeks of age for Exp.1 is shown in Table 9 and Table 10. The transition from 10.0 to 13.1 MJ/kg AME_n_ (calculated) decreased daily FI by 26.3% (130.35 versus 103.1 g/d; *p* < 0.01) and improved FCR by 12.2% (1.876 versus 2.137 g feed/g/d; *p* < 0.01) in a linear manner. The main effect of AME_n_ level had no significant effect on hen day production, egg weight, egg output or daily AME_n_ intake (Table 10). The increase in dietary SID lysine (calculated) from 4.8 to 7.8 g/kg resulted in an increase in EW of 4.6% (59.47 versus 62.21 g/egg; *p* < 0.05), but EO increased by 15.6% (48.91 versus 56.53 g/d) and subsequently improved FCR by 18.4% (2.435 versus 2.071; *p* < 0.01). Significant interactions between dietary AME_n_ and SID Lys were observed for EO *(p* = 0.024), FI *(p* = 0.028) and AME_n_ intake (*p* = 0.015) (Table 10).

### 3.2. Experiment 2

The analysed composition of the four basal diets used in Exp.2 is shown in Table 5. While CP and total AA values were in close agreement with the calculated values, the AME_n_ values determined using NIR technology were lower than the formulated values. These values were determined using the proximate analyses and then applying the WPSA equations [19]. Although this methodology is promising, accurate predictions are still elusive [22]. The values derived in this instance are likely incorrect when considering how accurate the AA analysis was.

The effects of dietary treatments on EW, HD production and FI for the period 27 to 30 weeks of age are shown in Table 11. The transition from 11.0 to 12.5 MJ/kg dietary AME_n_ decreased daily FI by 10.3% (105.5 versus 117.6 g/d; *p* < 0.01) and improved FCR by 12.2% (1.88 versus 2.14 g feed/g egg/d; *p* < 0.01) in a linear manner. It did not affect energy intake. SID Lys intake declined by 12.5% (885 versus 787 mg/d; *p* < 0.01) as dietary energy levels increased, but the increase in dietary SID Lys 6.0 to 9.0 g/kg increased daily SID Lys intake by 46.5% (678 versus 989 mg/d; *p* < 0.01). This resulted in an increase in EW of 3.4% (56.9 versus 58.8 g/egg; *p* < 0.05) and subsequently improved FCR by 5.99% (2.05 versus 1.93; *p* < 0.01). An interaction (*p* < 0.05) between dietary AME_n_ and SID Lys was observed for HD production only.

### 3.3. Comparison between Experiments

A comparison of the production parameters for each flock can be gained from the footnotes in Table 9 and Table 11 for Exp.1 and Exp.2, respectively. These values represent the output from all birds. The values shown in Table 12 represent only those birds included for modelling purposes after some hens were excluded because their FCR fell outside of the specified range. The MS hens had a lower BW, reduced EW and an increase in EO.

Although bird age differed between the two experiments by ten weeks, the results are still insightful. MS hens were more variable than HS hens for all parameters measured. On average, the HS birds were 185 g heavier than MS hens (*p* < 0.001) and gained more weight at 1.3 g/d compared to the 0.2 g/d for the MS hens *(p* < 0.001). There was a difference (*p* < 0.001) in FI between the strains, which was to be expected due to differences in BW. It is perhaps noteworthy that the FI for specific individuals in both experiments ranged from below 70 g/d to values that exceeded 150 g/d. The HD for HS hens was lower than that achieved by MS (85.7% versus 97.2; *p* < 0.001), but EW was heavier (61.6 g versus 58.1 g; *p* < 0.001). The disparities in age may have exacerbated these differences. The difference in FCR between the two strains was as expected, with 2.25 (g feed/g egg) measured in the HS hens and 2.00 (g feed/g egg) for the MS hens (*p* = 0.005).

**Figure 1:**
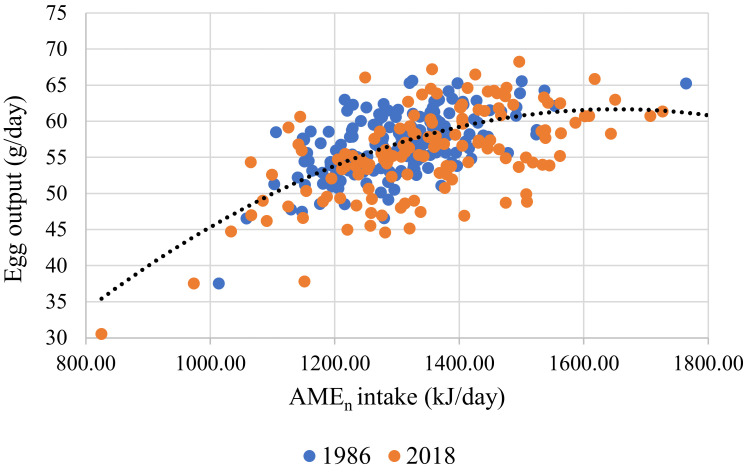
Response curve illustrating the relationship between individual hen egg output (g/day) and daily dietary AME_n_ intake (mg/day) for experiments conducted in 1986 and 2018. The fitted line for both genotypes, in black, is described by the relationship y = −34.507 + 0.111x − 3.2 × 10^−^^5 × 2^ (r^2^ = 0.374; *p* < 0.001). All birds with FCR values of less than 1.5 or higher than 2.4 were excluded.

**Figure 2:**
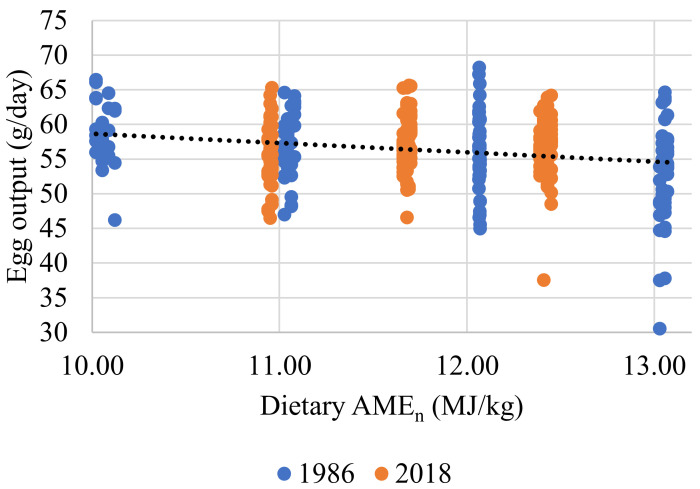
Response line illustrating the relationship between individual hen egg output (g/day) and dietary AME_n_ level (MJ/kg) for experiments conducted in 1986 and 2018. The fitted line for both genotypes, in black, is described by the relationship y = 71.138 − 1.314x + 0.567 × Modern strain (r^2^ = 0.052; *p* < 0.001). All birds with FCR values of less than 1.5 or higher than 2.4 were excluded.

**Figure 3:**
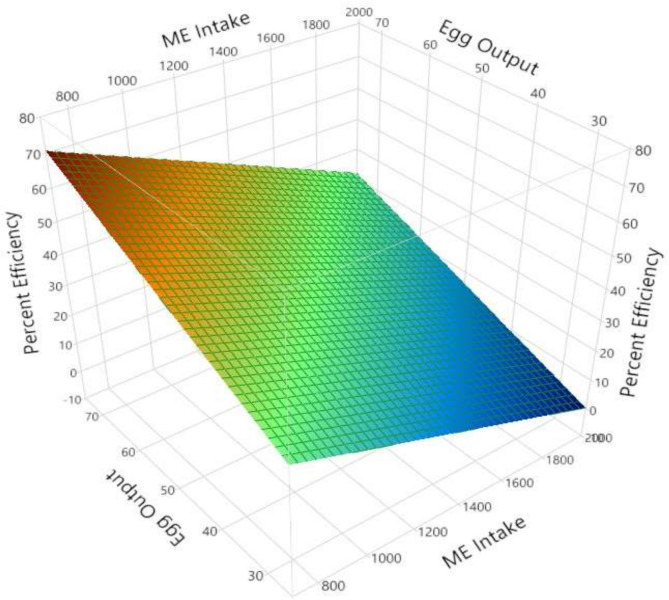
Surface response illustrating the relationship between the efficiency of AME_n_ utilization (%), individual hen egg output (g/day) and daily dietary AME_n_ intake (mg/day) for experiments conducted in 1986 and 2018. The response is described by the relationship y = 37.399 − 0.028x + 0.696z (r^2^ = 0.987; *p* < 0.001). All birds with FCR values of less than 1.5 or higher than 2.4 were excluded.

AME_n_ intake was strongly correlated with BW and EO (r = 0.557; *p* < 0.001). There were no significant differences between the HS and MS strains (Equation (1)). The relationship between EO and AME_n_ intake was significant (r = 0.374; *p* < 0.001). There were significant differences between the two strains (*p* < 0.001), with MS producing more grams of EO per kJ of AME_n_ intake (Equation (2) and Figure 1). The relationship between EO and the dietary levels of AME_n_ was weak, although significant (r = 0.052. *p* < 0.001). Strain played a significant role in this relationship (Equation (3) and Figure 2). When AME_n_ efficiency was considered as a percentage of utilization, a high degree of correlation was measured (r = 0.987; *p* < 0.001). The strain was not significant, but EO *(p* < 0.001) and AME_n_ intakes (*p* < 0.001) were (Equation (4) and Figure 3). The predictions for FI (r = 0.716; *p* < 0.001) and FCR (r = 0.588; *p* < 0.001) were strong and all production parameters were significant. The strain had no significant impact on either FI or FCR (Equations (5) and (6)).

**Figure 4:**
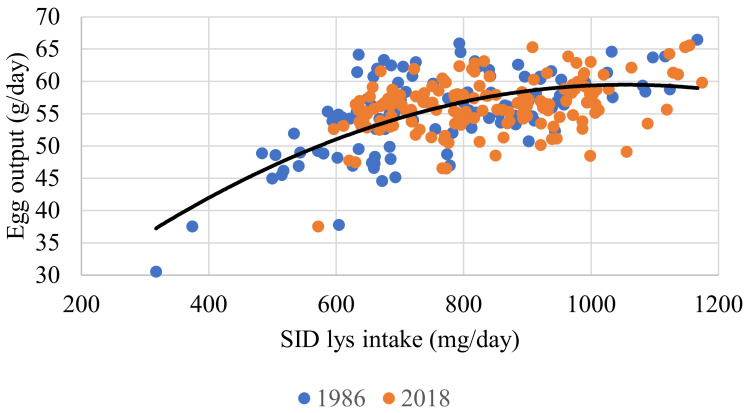
Response curve illustrating the relationship between individual hen average egg output (g/day) and daily SID Lys intake (mg/day) for experiments conducted in 1986 and 2018. The fitted line for both genotypes, in black, is described by the relationship y = 19.911 + 0.0760x − 36 × 10^−^^5 × 2^ (r^2^ = 0.299; *p* < 0.001). All birds with FCR values of less than 1.9 or higher than 2.4 were excluded.

**Figure 5:**
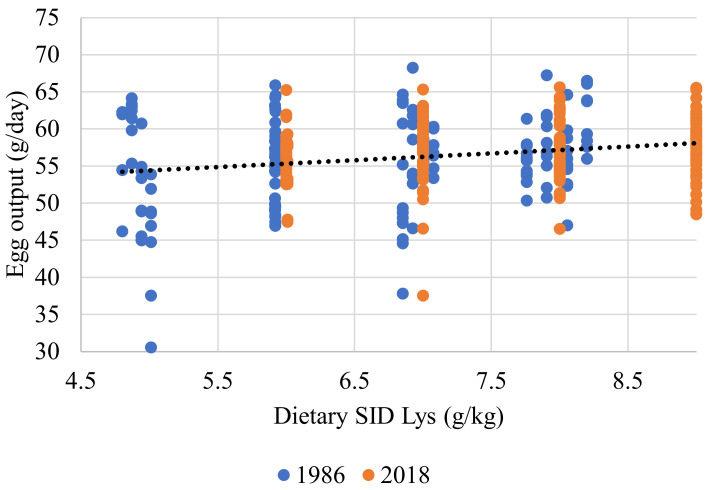
Response curve illustrating the relationship between individual hen egg output (g/day) and dietary SID Lys (g/kg) for experiments conducted in 1986 and 2018. The fitted line for both genotypes, in black, is described by the relationship y = 49.822 + 0.918x (r^2^ = 0.042; *p* < 0.001). All birds with FCR values of less than 1.5 or higher than 2.4 were excluded.

**Figure 6:**
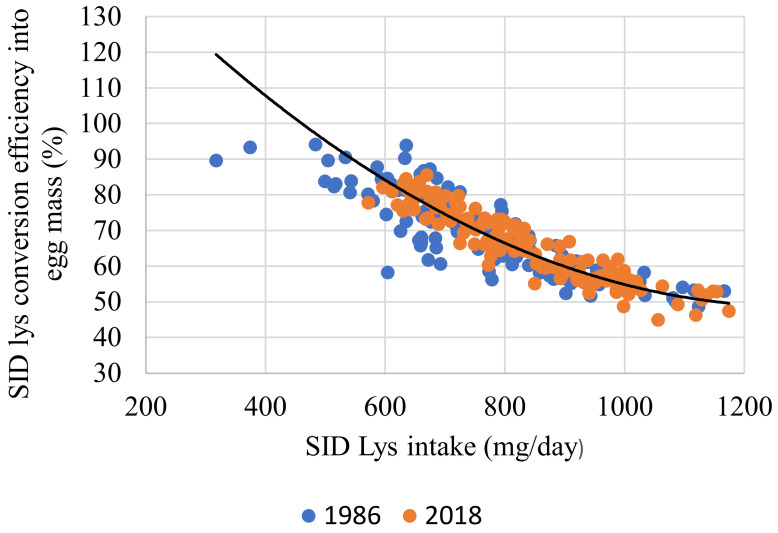
Relationship between dietary SID Lys intake (mg/day) and dietary SID Lys conversion efficiency into egg mass (%) of individual hens based on experiments conducted in 1986 and 2018. The SID Lys efficiency was calculated as (egg mass (g/day) × 9.3)/SID lys intake (mg/d) × 100. The fitted line for both genotypes, in black, is described by the relationship y = 73.626 − 0.084 SID Lys intake (mg) +1.083 Egg output (g) − 0.625 Modern strain (r^2^ = 0.950; *p* < 0.001). All birds with FCR values of less than 1.5 or higher than 2.4 were excluded.

When considering EO in response to SID Lys, the relationship was significant (r = 0.299; *p* < 0.001). In this instance, strain was not significant (Equation (8) and Figure 4). Although significant, the relationship between EO and the dietary SID Lys (g/kg) was far weaker (r = 0.042; *p* < 0.001) and the impact of strain was not significant (Equation (9) and Figure 5). SID Lys utilization, as a percentage, relative to SID Lys (mg/d) intake was significant (r = 0.950; *p* < 0.001). Body weight was not significant, but both EO and strain were, with the MS strain being marginally less efficient (Equation (10) and Figure 6).

## 4. Discussion

This study investigated and compared how individually housed brown laying hens of two different genotypes and age reacted to incremental levels of dietary AME_n_ and SID Lys. Rather than simply comparing the performance of the two strains, the objective was to evaluate the requirements and utilization of AME_n_ and SID Lys in HS and MS hens. When feeding individual hens, the degree of heterogeneity (brought about by measuring individual birds rather than the average of a group of hens) makes it challenging to estimate nutritional responses [23]. However, it was possible to develop response curves for both attributes, although the fit was reasonably low (r = 0.374 for AME_n_; r = 0.299 for SID Lys) (Figure 1 and Figure 4).

This discussion would not be complete if the benefits and drawbacks of working with individually housed hens were not considered. Influences on EO can be divided into internal factors linked to the genetic structure of the bird and external factors such as seasonal effects, stress and housing systems [24]. The advantage of housing and measuring individual hens is that outcomes are not blurred by averaging the measurements from two or more individuals, giving rise to a more accurate measurement of the internal factors. In contrast, the social and spatial constraints between hens living in a colony of cohorts are mainly absent. It is likely that under commercial, particularly cage-free conditions, FI is limited by this social interaction. High stocking densities exacerbate the problem of access to feed. This may explain why diets with higher nutrient densities sometimes lead to improved performance [25,26,27]. Pottgüter [28] reinforced this view, finding that modern genotypes of laying hens can cope well under different management systems, provided they are permitted to consume adequate amounts of feed. Conversely, individually housed hens will likely be less active [29] and tend to have improved feather cover, impacting energy requirements.

In order to determine the requirements for AME_n_ and SID Lys, flocks of hens, split into replicates comprising colonies of varying size, are fed graded levels of the component being tested. Requirements are then estimated for maintenance and production using regression analysis. These requirements are static [30] and need to be transposed into optimal doses for specific flocks. In order to estimate dietary concentrations (feed specifications) that will maximize returns, differing environmental, management and economic circumstances all need to be considered [29,31,32] together with the derived response data. Individual hens do not ‘respond’ to dietary levels of energy (AME_n_) and balanced protein (SID Lys). Instead, they adjust FI to consume adequate AME_n_ or SID Lys, whichever is first limiting (Equation (5)). Broadly, it is not easy to develop realistic responses that relate flock response to feed composition, and knowing which mathematical model is the most appropriate to use is a challenge [29,31,32]. In this study, the AME_n_: SID Lys ratio varied between treatments, the hens were of a different strain and age. The variability between individuals is higher than between groups of hens. Thus, the responses would be expected to be less well-defined (Equations (2) and (7) in Table 8 and Figure 1 and Figure 4).

**Figure 7:**
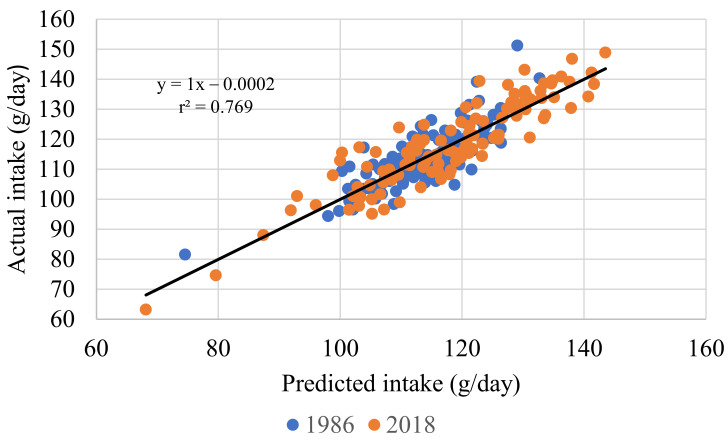
A scatter plot illustrating the relationship for individual hens between predicted feed intake (g/day) and actual feed intake (g/day) for experiments conducted in 1986 and 2018. The fitted line for both genotypes, in black, is described by the relationship y = 0.0002 + 1x (r^2^ = 0.769; *p* < 0.001). All birds with FCR values of less than 1.9 or higher than 2.4 were excluded.

Many factors will likely impact energy and nutrient utilization. These would include the physical form of the feed (the grist), how the birds were reared and managed prior to the experiment and climate control. It was not possible to control for these variables in the experiments reported here. However, the fact that the FI could be accurately predicted (r^2^ = 0.716) across both flocks of hens, consuming a range of diets, with a degree of accuracy (Equation (5) and Figure 7) would indicate that not controlling for these factors was perhaps of lesser importance.

### 4.1. Energy

In both experiments, the hens responded to incremental increases in AME_n_ by reducing FI intake such that their energy requirements were met (Table 10 and Table 11). There were no differences between the strains, and it was only BW and EO that impacted energy intake (Equation (1)). The view of the NRC [33] that requirements remain unchanged regardless of FI is supported here. This finding is reinforced by the fact that AME_n_ intake did not vary with dietary AME_n_ levels, regardless of hen genotype or age (Equation (4) and Figure 2). If indeed there was any impact of dietary energy level on EO, it would appear that high energy diets may have suppressed EO slightly. The declining SID Lys intakes may explain this observation as dietary AME_n_ levels increased (Table 6 and Table 9). Of note is that EW did not decline, confirming the observations of Morris and Gous [34] that when protein intakes drop below the optimum, egg number is more impacted upon than egg size.

The effect of AME_n_ on FI has presented conflicting results in the literature. Some observations indicate that hens can manage their energy intake by adjusting their feed intake [34,35]. Others suggested that some modern genotypes cannot regulate their FI due to changing dietary energy levels [27]. A meta-analysis conducted on 17 experiments [25] showed that energy consumption is significantly higher when hens were offered more concentrated feeds (on average, 3% per MJ/kg AME_n_). Pérez-Bonilla, Novoa, García, Mohiti-Asli, Frikha and Mateos [26] found that in hens housed in colonies of 13 hens in enriched cages, an increase in dietary energy led to an increase in energy intake. Regardless, it would appear that improved genotype, MS hens, have not lost their ability to regulate their energy intake.

Gous, et al. [36] stated that although dietary energy does not influence EO directly, it has an indirect effect due to its impact on FI and AA intake. The results of this study partially agree with this finding. In some instances, the birds may not have consumed enough feed to ensure an adequate AA intake. In Exp.1, there was a negative effect in HD production when birds were offered the diet containing the lowest level of SID Lys (5 g/kg) and the highest AME_n_ (13 MJ/kg). It is proposed that the balance between AME_n_ and SID Lys was such that the birds could not consume sufficient protein. In Exp.2, HD production was suppressed on the least dense diet (11 MJ/kg AME_n_ and 6 g/kg SID Lys), suggesting that the hens could not consume enough feed, thus depriving them of both protein and energy. Both of these diets would be outside of the range of diets used commercially.

In the experiments reported here, examining the main effect of dietary AME_n_ level in Exp.1 shows no meaningful effect on EW (Table 10). In contrast, in Exp.2, the birds offered the intermediate energy level diet (11.75 MJ/kg) produced significantly larger eggs. This can be partially explained by the reduction in SID Lys intake recorded at the higher energy levels in Exp.2. Bouvarel, Nys and Lescoat [25] found that mean EW increased with energy intake, whereas Pérez-Bonilla, Novoa, García, Mohiti-Asli, Frikha and Mateos [26] could not demonstrate any increase in egg size as dietary energy levels increased.

One of the critical components of this study was to examine the efficiency of energy utilization for EO between the two genotypes studied. Accordingly, the percentage of energy utilization per gram EO was calculated. From Equation (1), it was shown that each gram of EO required 9.157 kJ of AME_n_. This figure was used to calculate the proportion of energy contained in EO, versus the energy intake. The value used per g of EO is in reasonable agreement with the values of 8.4, 7.5, 8.66 and 10 kJ/g of egg output proposed by Emmans [37], NRC [33], Chwalibog [38] and Sakomura, Reis, Ferreira and Gous [39], respectively. The model generated was extremely strong (r^2^ = 0.987), with AME_n_ intake and EO being highly significant. Notably, there were no differences in energy utilization between the two strains, bearing in mind that any differences in BW were accounted for by the model used. However, it can be seen from Figure 3 that the efficiency of energy utilization improved as EO increased or when AME_n_ intake decreased, similar to the finding of Peguri and Coon [40]. From a maintenance perspective, the energy requirement was 352.3 kJ/kg BW. For a hen of 1960 g, this translates into a figure of 415.5 kJ/kg BW^0.75^, a value that compares with the 360.0 kJ/kg^.75^ [41], 540.0 kJ/kg^0.75^ [33], 400.0 kJ/kg^0.75^ [38] and 472.3 kJ/kg^0.75^ [42].

There were significant interactions between dietary AME_n_ and SID Lys. In Exp.1 this can partially be explained by the fact that hens with a high BW require more energy for maintenance, and in the process of increasing energy intake, they also consume a surfeit of SID Lys. An examination of the data shown in Table 9 may offer a second explanation. Those birds on diets with the highest AMEn level (13 MJ/kg) but with the lowest SID Lys level (5 g/kg) may not have consumed enough SID Lys to maintain normal production levels. In Exp.2, those hens that consumed the diet with the lowest nutrient density (11 MJ/kg AMEn; 6 g/kg SID Lys) appeared to be unable to consume enough energy and nutrients to meet requirements (Table 11).

### 4.2. Amino Acids

The description of the AA responses used and the nutrient requirement determined through their use are complex. There were significant differences between the two flocks for SID Lys intake (Equation (7)). The difference between strains was slight and may partially be ascribed to the fact that some of the diets used in Exp.1 were very low in SID Lys. This contention is supported to some extent by the fact that the SID Lys intake required to support EO did not differ between strains (Equation (8) and Figure 4). The correlation between EO and dietary SID Lys content was poor; nevertheless it was significant. Egg output tended to increase at higher dietary SID Lys levels (Equation (9) and Figure 5), as widely reported [13,31,32]. The analysed composition of the four basal diets used in Exp.1 is shown in Table 2. It was found that analysed values for both the Lys and TMEn levels were in close agreement with the formulated values. However, the determined methionine values were below expectation.

Upon evaluating the impact of genotype on the utilization of SID Lys, it was found that strain had a small but significant impact on efficiency (Equation (10) and Figure 6), with the MS being marginally better. Efficiency in SID Lys utilization decreased as EO increased production levels, or when dietary SID Lys content was higher, which concurs with the findings of Spek [21]. In this experiment, the SID Lys requirement per gram EO was 14.2 mg/g. This is somewhat higher than the value of 9.45 [43] but more in line with Rostagno, Albino, Hannas, Donzele, Sakomura, Perazzo, Saraiva, de Abreu, Rodrigues, de Oliveira, Barreto and Brito [39] of 12.4 mg/g.

It is intimated that the protein requirements of modern genotypes have increased because they have a higher EO [12,13,14,15,16]. Modern laying genotypes have a lower BW with a reduced EW [2]. Egg output increases because hens lay longer clutches (fewer pause days) and not because EW is higher. In reality, EO on any particular day does not increase. Regardless of how many eggs hens might lay in their lifetime, they still fulfil their nutrient requirements daily. Thus, it is likely that the absolute nutrient requirement of MS laying hens has decreased. Coupled with the finding that the MS utilizes SID Lys with marginally greater efficiency, it is unlikely that modern genotypes have a higher protein requirement.

### 4.3. Feed Intake

The prediction of FI is central to the practical feeding of laying hens. Although the FI of the MS hens was 4.5 g/d lower than the HS hens (112.5 versus 117.0 g; *p* < 0.001), a single model could be used to describe FI (Equation (1) and Figure 7). Some HS hens were offered a diet containing only 10.1 MJ/kg of AME_n_, with high fibre content, and this may have resulted in an exaggerated FI for those individuals. It is generally assumed that a hen’s daily requirements for nutrients remain unchanged by FI [35]. It is also widely believed that “birds eat to satisfy their energy requirements”, but this view is too simplistic and of limited value in predicting FI [43]. Leeson and Summers [44] contended that there is little evidence to suggest that the hen can adjust FI with enough precision to maintain a consistent intake of nutrients other than energy. When evaluating the response of individual hens, both AME_n_ and SID Lys, along with the BW and EO of the hen, had a significant impact on FI (Equation (5)). Thus, the FI of individually housed hens is determined by the first limiting component of the diet, be that protein or energy. This does not change with age or as production parameters improve. However, when birds are housed in colonies, the adjustment of FI to meet requirements may not be possible. Bird genotype had no impact on the hen’s ability to adjust FI, suggesting that the underlying metabolism of laying hens has not changed as genotypes have improved. A combination of low FI and a higher EO improved FCR by 12.5% (1.95 versus 2.25; *p* < 0.001) in the MS flock.

### 4.4. Genotype

The experiments reported here allowed for an evaluation of how the genotype of brown laying hens has evolved over three decades. At a phenotypic level, there are differences between the HS and MS hens. The results of this study highlighted that hen response, in terms of EO, has remained similar with only a trend (*p* = 0.091) towards greater EO in the MS. From Table 12, it is evident that the BW of MS hens has decreased which is a similar finding reported by several authors [2,5,45]. The MS hens displayed higher HD production, lower EW but increased EO simply because they laid more eggs

Historically, it has been assumed that hens gain weight throughout the laying cycle. Emmans [37] made allowance for a gain of one gram per day, whereas Leeson and Summers [44] allowed for 2 to 3 g of gain in brown hens aged 25 to 30 weeks and Rostagno et al. [42] made provision for a gain of 0.4 g/d. The HS hens gained 1.3 g/d during the experimental period, whereas the MS birds gained only 0.2 g/d. This is an important difference because historically, the steady weight gain with age in laying hens lead to a gradual increase in FI. This would appear not to be the case in MS hens, a factor that will impact practical feeding systems. As expected, the EO was higher in the case of the MS birds. EW was lower, but due to higher HD production, MS hens had a higher EO (56.5 versus 53.1). This finding concurred with previously reported values [6].

Selection pressure to reduce EW [6] might result in reduced gains in EO in response to increased protein intakes than was previously the case. This was borne out by the results from Exp.2, where for the MS hens, EW increased by 0.65 g per 100 mg of SID Lys consumed. This figure was higher at 0.80 g per 100 mg of SID Lys consumed for the HS strain. Both of these values are lower than the 1.19 g, 1.59 g and 1.42 g per 100 mg of SID Lys intake, calculated from the work of [14,32,46], respectively.

## 5. Conclusions

The HS and MS strains used in these experiments differed in age, body weight, egg weight, hen day production and egg output. Both HS and MS hens were able to regulate their FI such that their energy intake remained constant. Thus, FI intake and FCR could be predicted using the same relationship for both strains. Modern laying hen genotypes have not lost the ability to maintain a consistent energy intake by adjusting FI when dietary AME_n_ level varies. Energy utilization was the same for both genotypes, with efficiency improvements at lower EO and energy intakes. The efficiency of protein utilization, as measured in terms SID Lys utilization, has not declined in the improved genotype. Instead, efficiency would appear to have improved marginally. The AME_n_ and SID Lys requirements for modern layer genotypes for maintenance and egg production have not changed over their heritage predecessors.

## Figures and Tables

**Table 1:** Composition and the diets used in 1986 Experiment 1 on an as-fed basis.

Feed Ingredient, g/kg	I	II	III	IV
Yellow maize	506.2	377.4	681.4	522.4
Wheat bran	197	200	45	46
Rice bran	–	60.0	–	–
Fishmeal	20	20	20	20
Cane molasses	25	–	–	–
Soybean meal	73	204	–	–
Full fat soybeans	–	–	123	282
Sunflower husk	75	35	–	–
Acid oil	–	–	25	25
DL-methionine	0.32	0.12	0.11	0.15
Salt	4	4	4	4
Limestone powder	87	88	88	88
Monocalcium phosphorus	10	9	11	10
Vitamin and mineral premix	2.5	2.5	2.5	2.5
Total	1000	1000	1000	1000

**Table 2:** Formulated, recalculated and analysed nutrient composition of the Experimental basal diets on an as-fed basis for Experiment 1.

Exp.1 Calculated Nutrient, g/kg	I	II	III	IV
TME_n_ (MJ/kg)	10.6	10.6	13.1	13.08
Crude protein	122	180	125	173
Total lysine	5.32	9.13	5.63	8.91
**Recalculated AME_n_, MJ/kg, Standardized Ileal Digestible Amino Acids, Minerals, Lipid and Fibre, g/kg**				
AME_n_	10.1	10.0	13.0	13.1
Crude protein	121	177	119	163
Lysine ^1,2^	4.80	8.20	5.01	7.76
Methionine	2.23	2.76	2.13	2.62
Methionine + Cysteine	3.92	5.09	3.84	4.72
Isoleucine	4.06	6.58	4.33	6.35
Tryptophan	1.06	1.76	1.05	1.65
Threonine	3.61	5.60	3.80	5.31
Valine	4.79	6.63	5.14	6.86
Arginine	6.39	10.51	6.44	9.72
Calcium	35.1	35.4	35.1	35.3
Available phosphorus	3.11	3.33	3.15	3.26
Sodium	1.96	1.93	1.79	1.81
Crude lipid	32.9	37.4	77.4	99.7
Crude fibre	73.4	62.9	28.5	32.4
**Analysed TME_n,_ MJ/kg and Total Nutrients, g/kg ^3^**				
TME_n_	11.0	11.0	13.0	13.2
Crude protein	130	189	120	153
Lysine	5.10	8.50	4.90	7.20
Methionine	1.90	2.48	1.90	3.00
Isoleucine	4.60	7.40	4.40	6.00
Threonine	4.20	6.70	3.90	5.00
Valine	5.50	8.80	5.20	6.80
Arginine	6.10	11.50	5.90	8.00
Calcium	32.0	32.4	34.2	31.6
Phosphorus	5.50	7.20	5.60	6.00

^1^ Total amino acid: ^2^ The ideal amino acid profile used was Lys, 100; Met, 45; Met + Cys, 82; Thr, 72; and Trp, 20; ^3^ As measured by the laboratory of the UKZN.

**Table 3:** Composition of Experimental diets, blend proportions and recalculated nutrient levels of basal diets for Experiment 1.

Basal Feed Blends (g/kg)			Basal Feed Blends, g/kg				Calculated Nutrient Levels	
Feed Number	TME_n_, MJ/kg ^1^	Total Lysine, g/kg ^1^	I	II	III	IV	AME_n_, MJ/kg ^2^	SID Lys, g/kg ^2^
1	10.0	5.0	1000	–	–	–	10.12	4.80
2	10.0	6.0	670	330	–	–	10.09	5.92
3	10.0	7.0	330	670	–	–	10.05	7.08
4	10.0	8.0	-	1000	–	–	10.02	8.20
5	11.0	5.0	670	–	330	–	11.08	4.87
6	11.0	6.0	450	220	220	110	11.06	5.92
7	11.0	7.0	220	450	110	220	11.04	7.00
8	11.0	8.0	-	670	–	330	11.03	8.05
9	12.0	5.0	330	–	670	–	12.07	4.94
10	12.0	6.0	220	110	450	220	12.07	5.92
11	12.0	7.0	110	220	220	450	12.07	6.93
12	12.0	8.0	-	330	–	670	12.06	7.91
13	13.0	5.0	–	–	1000	–	13.03	5.01
14	13.0	6.0	-	–	670	330	13.04	5.92
15	13.0	7.0	–	–	330	670	13.06	6.85
16	13.0	8.0	–	–	–	1000	13.07	7.76

^1^ When the diets were originally formulated, it was done on the basis of TME_n_ and total lysine; ^2^ These values represent the recalculated values for each diet using updated matrix values for AME_n_ and SID Lys.

**Table 4:** Composition of the basal diets used in Experiment 2 (raw material crude protein in %) for Experiment 2.

Feed Ingredient, g/kg	I	II	III	IV
Yellow maize 7.1%	524	464	695	511
Wheat middlings 15%	189	75	–	–
Soybean meal 46%	78	252	136	284
Sunflower meal 35.5%	100.0	100	50.0	50.0
Soya oil	5.0	5.00	14.8	45.8
Limestone	90	89	89	89
Monocalcium phosphate	5.4	4.6	7.1	5.6
Salt	3.0	3.0	3.2	3.1
BioLysine	1.70	–	0.9	–
DL-Methionine	1.10	2.40	1.29	2.80
L-Threonine	0.16	0.09	0.11	0.33
L-Valine 10%	–	2.17	–	6.06
Layer premix ^1^	2.50	2.50	2.50	2.50
Phytase 1200 FYT ^2^	0.06	0.06	0.06	0.06
Total	1000	1000	1000	1000

^1^ The premix supplied per tonne: 8.0 MIU Vit A, 3.0 MIU Vit D, 20.0 g Vit E, 3.0 g Vit K, 35.0 g nicotinic acid, 12 g pantothenic acid, 1 g folic acid, 6 g riboflavin, 0.02 cyanocobalamin, 0.10 g biotin, 5.0 g pyridoxine, 2.0 g thiamine, 8.0 g copper, 0.20 g cobalt, 0.50 g molybdenum, 1.0 g iodine, 0.30 g selenium, 60.0 g iron, 60.0 g zinc, 90.0 g manganese, 20.0 g Oxicap E2 (antioxidant); ^2^ Matrix values for phytase (DSM HiPhos GT 10,000, 1200 FYT) were: 2.5% P avail., 2.8% Ca, 690,000 kcal/kg AME_n_, 240% lysine, 72% methionine, 210% methionine1cystine, 214% threonine, 174% isoleucine, 64% tryptophan, 212% valine, and 204% arginine with amino acids on a digestibility basis.

**Table 5:** Formulated and analysed nutrient composition of the basal diets on an as-fed basis for Experiment 2.

Nutrients, g/kg	I	II	III	IV
AME_n_, MJ/kg	10.94	10.96	12.39	12.50
Crude protein	142	200	135	189
Total Lysine	6.75	10.07	6.58	9.98
Total Methionine + Cysteine	6.07	8.91	5.89	8.78
Total Threonine	5.41	7.55	5.02	7.46
Standardized ileal digestible amino acids, Ca, P, Fat, Fibre and Na, g/kg				
Lysine ^1,2^	6.01	9.01	6.01	9.00
Methionine	3.36	5.42	3.44	5.56
Methionine + Cysteine	5.41	8.12	5.43	8.10
Isoleucine	4.81	7.73	4.98	7.53
Tryptophan	1.34	2.07	1.24	1.94
Threonine	4.43	6.59	4.38	6.57
Valine	5.76	8.57	5.72	8.55
Arginine	8.53	12.84	7.88	11.91
Calcium	35.0	35.0	35.0	35.0
Available phosphorus	3.50	3.50	3.50	3.50
Sodium	1.78	1.77	1.77	1.78
Fat	37.5	34.0	46.3	72.3
Crude fibre	53.9	46.1	31.6	31.3
Analysed total nutrients, g/kg ^3^				
AME_n_ (MJ/kg) ^3^	10.86	10.51	11.35	11.70
Crude protein	148	199	137	194
Lysine	6.76	10.09	6.45	9.84
Methionine	3.05	5.34	3.39	5.99
Methionine + Cystine	6.05	8.29	5.57	8.66
Threonine	5.15	7.43	5.09	7.39

^1^ SID = Standardised ileal digestibility; ^2^ The ideal amino acid profile used was Lys: 100; Met: 50; Met + Cys: 90; Ile: 68; Thr: 68; Trp: 20; and Val: 88; ^3^ Determined using near infrared (NIR) technology.

**Table 6:** Composition of Experimental diets, blend proportions and nutrient levels of basal diets Experiment 2.

Formulated Nutrient Levels			Basal Feed Blends, g/kg			
Feed Number	AME_n_, MJ/kg	Digestible Lysine, g/kg	I	II	III	IV
1	11.00	6.0	1000	–	–	–
2	11.00	7.0	667	333	–	–
3	11.00	8.0	333	667	–	–
4	11.00	9.0	-	1000	–	-
5	11.75	6.0	500	–	500	-
6	11.75	7.0	335	165	335	165
7	11.75	8.0	165	335	165	335
8	11.75	9.0	–	500	-	500
9	12.50	6.0	-	–	1000	–
10	12.50	7.0	–	-	667	333
11	12.50	8.0	–	–	333	667
12	12.50	9.0	-	–	-	1000

**Table 7:** Prediction of AME_n_ intake (kJ/d) egg output response to AME_n_ intake, efficiency of AME_n_ utilization, feed intake (g/d) and FCR, g feed/g egg for both Experiments combined using linear regression (*n* = 326 measurements with 28 diets).

Equation	Dependent Variable	Independent Variable	Parameter Estimate	Standard Error	*p* Value
1	AME_n_ intake, kJ/d (r^2^ = 0.557)	Intercept	121.279	60.789	0.047
		Body weight, g	0.352	13.810	<0.001
		Egg output, g/d	9.157	0.959	<0.001
2	Egg output, g/d (r^2^ = 0.374)	Intercept	−34.507	14.090	0.015
		AME_n_ intake, kJ/d	0.111	0.021	<0.001
		AME_n_ intake, kJ/d	−3.2 × 10^−^^5^	7.8 × 10^−^^6^	<0.001
		Strain ^1^	1.911	0.478	<0.001
3	Egg output, g/d (r^2^ = 0.052)	Intercept	71.138	3.997	<0.001
		AME_n_, MJ/kg	−1.3144	0.338	<0.001
		Strain	1.122	0.567	=0.049
4	AME_n_ utilization, % (r^2^ = 0.987)	Intercept	37.399	0.264	<0.001
		AME_n_ intake, kJ/g d	−0.028	0.000	<0.001
		Egg output g/d	0.696	0.005	<0.001
5	Feed intake, g/d (r^2^ = 0.716)	Intercept	112.633	7.745	<0.001
		Body weight, g	0.026	2.130	<0.001
		Egg output, g/d	0.957	0.082	<0.001
		SID Lys, g/kg	−1.703	0.327	<0.001
		AME_n_, MJ/kg	−7.762	0.470	<0.001
6	FCR, g feed/g egg (r^2^ = 0.588)	Intercept	4.020	0.137	<0.001
		Body weight, g	4.5 × 10^−^^4^	0.038	<0.001
		Egg output, g/d	−0.019	0.001	<0.001
		SID Lys, g/kg	−0.029	0.006	<0.001
		AME_n_, MJ/kg	−0.135	0.008	<0.001

^1^ A correction factor to be applied to the MS hens.

**Table 8:** Prediction of SID Lys intake mgJ/d), egg output response to SID Lys intake and dietary level, and efficiency of SID Lys utilization for both Experiments combined using linear regression (*n* = 326 measurements with 28 diets).

Equation	Dependent Variable	Independent Variable	Parameter Estimate	Standard Error	*p* Value
7	SID Lys intake, mg/d (r^2^ = 0.285)	Intercept	−25.956	77.876	0.739
		Egg output, g/d	14.190	1.385	<0.001
		Strain ^1^	56.526	14.507	<0.001
8	Egg output, g/d (r^2^ = 0.299)	Intercept	19.911	4.992	<0.001
		SID Lys intake, mg/d	0.076	0.013	<0.001
		SID Lys intake mg/d	−3.6 × 10^−^^5^	7.7 × 10^−^^6^	<0.001
9	Egg output,g/d (r^2^ = 0.042)	Intercept	49.822	1.697	<0.001
		Dietary SID Lys, g/kg	0.918	0.235	<0.001
10	SID Lys utilization, % r^2^ = 0.950)	Intercept	73.626	1.541	<0.001
		SID Lys intake, mg/d	−0.084	0.001	<0.001
		Egg output, g/d	1.083	0.032	<0.001
		Strain	−0.625	0.294	0.034

^1^ A correction factor to be applied to the MS hens.

**Table 9:** Effects of dietary treatments on egg parameters, body weight (BW), feed intake (FI), feed conversion ratio (FCR), nitrogen-corrected apparent metabolizable energy (AME_n_), and standardized ileal digestible lysine (SID Lys) intake of Hisex Brown layers from 37 to 40 weeks of age for Experiment 1.

Treatment		Layer Performance							
AME_n_, MJ/kg	SID Lys, g/kg	Hen Day, %	Egg Weight, g	Egg Output, g/d	Feed Intake, g/d	Body Weight, g	FCR, g Feed/g egg	AME_n_ Intake, kJ/d	SID Lys Intake, mg/d
10.0 11.0 12.0 13.0	5.0 6.0 7.0 8.0 5.0 6.0 7.0 8.0 5.0 6.0 7.0 8.0 5.0 6.0 7.0 8.0	82.14 ^b,c^ 86.61 ^b,c,d^ 81.85 ^b^ 91.88 ^d^ 91.07 ^b,c,d^ 88.10 ^b,c,d^ 83.04 ^b,c,d^ 88.39 ^b,c,d^ 82.44 ^b,c,d^ 87.50 ^b,c,d^ 88.39 ^b,c,d^ 91.37 ^c,d^ 70.24 ^a^ 86.04 ^b,c,d^ 82.74 ^b,c,d^ 89.58 ^b,c,d^	59.28 62.74 58.77 64.08 60.94 62.07 61.70 62.81 58.83 63.53 63.98 60.14 59.20 62.18 63.60 61.85	50.93 ^b,c^^,d^ 54.36 ^b,c,d,e^ 49.35 ^b,c^ 58.97 ^e^ 55.84 ^d,e^ 54.53 ^b,c,d,e^ 51.22 ^b,c,d^ 55.42 ^c,d,e^ 48.32 ^b^ 55.60 ^c,d,e^ 56.49 ^d,e^ 56.11 ^d,e^ 41.60 ^a^ 53.30 ^b,c^^,d,e^ 52.43 ^b,c^^,d^ 55.41 ^c,d^^,e^	132.5 ^g^ 132.0 ^g^ 125.3 ^e,f^^,g^ 131.2 ^g^ 129.6 ^f,g^ 119.9 ^d,e^^,f^ 116.0 ^b,c^^,d,e^ 116.9 ^b,c^^,d,e^ 112.6 ^b,c^^,d^ 117.4 ^c,d^^,e^ 114.6 ^b,c^^,d^ 112.0 ^b,c^^,d^ 92.80 ^a^ 106.9 ^b,c^ 106.5 ^b^ 106.4 ^b^	2015 2092 2043 2048 2078 2054 2044 1993 2010 2181 2163 2129 1867 2066 2082 2117	2.739 ^g^ 2.452 ^f,g^ 2.680 ^g^ 2.239 ^b,c^^,d,e,f^ 2.352 ^d,e^^,f^ 2.201 ^a,b^^,c,d,e,f^ 2.329 ^c,d^^,e,f^ 2.110 ^a,b^^,c,d,e^ 2.371 ^e,f^ 2.133 ^a,b^^,c,d,e^ 2.049 ^a,b^^,c^ 2.029 ^a,b^ 2.253 ^b,c^^,d,e,f^ 2.011 ^a,b^ 2.056 ^a,b^^,c,d^ 1.917 ^a^	1341 ^b,c^^,d^ 1331 ^a,b^^,c,d^ 1260 ^a,b^ 1314 ^a,b^^,c,d^ 1436 ^d^ 1326 ^a,b^^,c,d^ 1281 ^a,b^^,c^ 1289 ^a,b^^,c^ 1360 ^b,c^^,d^ 1416 ^d^ 1382 ^b,c^^,d^ 1351 ^b,c^^,d^ 1209 ^a^ 1394 ^c,d^ 1390 ^c,d^ 1391 ^c,d^	636 ^c^ 782 ^e,f^ 887 ^g,h^ 1076 ^i^ 631 ^c^ 710 ^d^ 813 ^f^ 941 ^h^ 557 ^b^ 695 ^c,d^ 793 ^e,f^ 885 ^g,h^ 465 ^a^ 633 ^c^ 730 ^d,e^ 826 ^f,g^
SEM Significance (*p* =)		3.402 0.003	1.430 0.057	2.268< 0.001	3.775< 0.001	61.55 0.132	0.106< 0.001	46.350.027	4.26< 0.001

^a,b^^,c,d,e,f^ Means within columns not sharing a common superscript are significantly different at the 5% level of probability; Mean performance: Hen day 85.66%, Egg weight 61.59 g, Egg output 53.07, Feed intake 117.0, Body weight 2061 g, FCR 2.247; AME_n_ intake 1342 KJ/d; SID Lys intake 752 mg/d.

**Table 10:** Main effects of dietary treatments on egg parameters, body weight, feed intake, feed conversion ratio (FCR), nitrogen-corrected apparent metabolizable energy (AME_n_) and standardized ileal digestible lysine (SID Lys) intake of Hisex Brown layers from 37 to 40 weeks of age for Experiment 1.

Treatment		Layer Performance							
		Hen day, %	Egg weight, g	Egg output, g/d	Feed Intake, g/d	Body weight, g	FCR, g feed/g egg	AME_n_ intake, kJ/d	SID Lys intake, mg/d
Main effect: AME_n_ 10.0 11.0 12.0 13.0 Main effect:SID Lysine 5.0 6.0 7.0 8.0 Significance (*p* =) Dietary energy (AME_n_) Digestible lysine (Lys) AME_n_ × Lys interaction		85.49 87.65 87.43 82.07 81.16 ^a^ 86.98 ^b,c^ 84.04 ^a,b^ 90.40 ^c^ 0.094 0.002 0.132	61.16 61.88 61.62 61.70 59.47 ^a^ 62.71 ^b^ 61.93 ^b^ 62.21 ^b^ 0.942 0.009 0.153	53.28 54.25 54.13 50.63 48.91 ^a^ 54.46 ^b,c^ 52.34 ^b^ 56.53 ^c^ 0.105 <0.001 0.024	130.2 ^d^ 120.6 ^c^ 114.1 ^b^ 103.1 ^a^ 116.6 119.4 115.4 116.6 <0.001 0.577 0.028	2050 ^a,b^ 2042 ^a,b^ 2121 ^b^ 2032 ^a^ 1992 2093 2089 2071 0.018 0.461 0.914	2.534 ^c^ 2.248 ^b^ 2.145 ^a,b^ 2.061 ^a^ 2.435 ^c^ 2.206 ^b^ 2.278 ^a,b^ 2.071 ^a^ <0.001 <0.001 0.806	1312 1333 1377 1345 1334 1367 1327 1339 0.217 0.584 0.015	840 ^d^ 774 ^c^ 733 ^b^ 664 ^a^ 571 ^a^ 710 ^b^ 804 ^c^ 923 ^d^ <0.001 <0.001 0.141

^a,b^^,c,d^ Means within columns not sharing a common superscript are significantly different at the 5% level of probability; Mean performance: Hen day 85.66%, Egg weight 61.59 g, Egg output 53.07, Feed intake 117.0, Body weight 2061 g, FCR 2.247, AME_n_ intake 1342 KJ/d, SID Lys intake 752 mg/d.

**Table 11:** Effects of dietary treatments on egg parameters, body weight, feed intake, feed conversion ratio (FCR), nitrogen-corrected apparent metabolizable energy (AME_n_) and standardized ileal digestible lysine (SID Lys) intake of Hy-Line Brown layers from 27 to 30 weeks of age for Experiment 2.

Treatment		Layer Performance							
AME_n_, MJ/kg	Digestible Lysine, g/kg	Hen Day, %	Egg Weight, g	Egg Output, g/d	Feed Intake, g/d	Body Weight, g	FCR, g Feed/g egg	AME_n_ Intake, kJ/d	SID Lys Intake, mg/d
11.00 11.75 12.50	6.0 7.0 8.0 9.0 6.0 7.0 8.0 9.0 6.0 7.0 8.0 9.0	93.57 ^a^ 98.21 ^c^ 97.77 ^b,c^ 94.64 ^a,b^ 98.21 ^c^ 96.65 ^a,b,c^ 96.43 ^a,b,c^ 98.88 ^c^ 99.11 ^c^ 97.99 ^b,c^ 96.68 ^a,b,c^ 98.21 ^c^	56.13 ^a^ 57.37 ^a^ 58.21 ^a,b^^,c^ 58.41 ^a,b,c^ 57.64 ^a,b^ 58.43 ^a,b,c^ 60.29 ^c^ 60.04 b^c^ 56.81 ^a^ 58.33 ^a,b,c^ 57.09 ^a^ 58.00 ^a,b,c^	52.44 ^a^ 56.36 ^b,c^ 56.93 ^b,c^ 55.30 ^a,b^ 56.61 ^b,c^ 56.52 ^b,c^ 58.13 ^b,c^ 59.38 ^c^ 56.28 ^b,c^ 57.30 ^b,c^ 55.25 ^a,b^ 57.00 ^b,c^	114.7 ^c,d^^,e,f^ 121.1 ^f^ 117.4 ^e,f^ 117.0 ^e,f^ 115.5 ^d,e,f^ 112.6 ^c,d,e^ 114.9 ^c,d,e,f^ 110.4 ^b,c,d^ 108.9 ^b,c^ 105.6 ^a,b^ 105.4 ^a,b^ 102.1 ^a^	1847 1885 1851 1834 1844 1869 1914 1900 1865 1878 1922 1910	2.198 ^f^ 2.157 ^e,f^ 2.062 ^d,e^ 2.135 ^e,f^ 2.040 ^c,d,e^ 2.005 ^c,d^ 1.984 ^c,d^ 1.860 ^a,b^ 1.934 ^b,c^ 1.851 ^a,b^ 1.928 ^b,c^ 1.798 ^a^	1255 1325 1285 1283 1347 1315 1343 1292 1349 1311 1310 1272	689 ^a^ 847 ^d^ 939 ^e^ 1053 ^g^ 693 ^a,b^ 788 ^c^ 919 ^e^ 994 ^f^ 653 ^a^ 739 ^b^ 843 ^d^ 919 ^e^
SEM Main effect: AME_n_ 11.00 11.75 12.50 Main effect SID Lys 6.0 7.0 8.0 9.0 Significance *(p* =) ANOVA Dietary energy (AME_n_) Digestible Lysine (Lys) AME_n_ x Lys interaction		4.909 96.09 ^a^ 97.55 ^b,c^ 98.04 ^c^ 97.04 97.62 96.97 97.25 <0.010 0.070 0.903 <0.05	3.567 57.55 ^a^ 59.10 ^b^ 57.57 ^a^ 56.88 ^a^ 58.05 ^a,b^ 58.59 ^b^ 58.82 ^b^ 0.002 <0.05 <0.05 0.732	4.696 55.30 ^a^ 57.66 ^b^ 56.50 ^a,b^ 55.17 56.73 56.84 57.22 <0.001 <0.05 0.143 0.204	9.067 117.6 ^c^ 113.3 ^b^ 105.5 ^a^ 113.0 113.1 112.9 109.9 <0.001 <0.001 0.255 0.298	143.91 1854 1882 1893 1852 1877 1894 1881 0.095 0.290 0.527 0.839	0.174 2.137 ^c^ 1.972 ^b^ 1.876 ^a^ 2.054 ^b^ 2.004 ^b^ 1.994 ^a,b^ 1.931^a^ <0.001 <0.001 <0.01 0.114	27.89 1287 1324 1310 1318 1317 1313 1282 0.196 0.131 0.293 0.315	18.184 885 ^c^ 848 ^b^ 787 ^a^ 678 ^a^ 792 ^b^ 903 ^c^ 989 ^d^ <0.001 <0.001 <0.001 0.124

^a,b^^,c,d,e,f^ Means within columns not sharing a common superscript are significantly different at the 5% level of probability; Mean performance: Hen day: 97.22%; Egg weight: 58.08 g; Egg output: 56.49; Feed intake: 112.19; Body weight: 1876 g; FCR: 1.996; AME_n_ intake: 1307 KJ/d; SID Lys intake: 840 mg/d.

**Table 12:** A comparison of production parameters achieved by individually housed, brown laying hens in Experiments 1 and 2 (Experiment 1 *n* = 143; Experiment *n* = 182).

Parameter Measured	Historic Stain	Modern Strain	*p*-Value ^1^
Body weight, g	2058 ± 206	1873 ±142	<0.001
Change in weight, g ^2^	1.102 ±3.39	5.838 ± 1.445	<0.001
Egg weight, g	62.436 ± 4.159	58.207 ± 3.418	0.038
Hen day production, %	89.211 ± 8.671	97.684 ± 3.615	<0.001
Egg output, g/d	55.675 ± 6.215	56.88 ± 4.192	<0.001
Feed intake, g/d	115.701 ± 15.348	111.786 ± 9.562	0.005
FCR, g feed/g egg	2.082 ± 0.190	1.971 ± 0.165	0.005

^1^*p*-value represents the differences between strains; ^2^ The change in body weight over the 28 ds data collection period.

## Data Availability

Data is available upon request from the corresponding author.
